# Supplementary material for: Restraint Use for Child Occupants in Dubai, United Arab Emirates
Source: Int J Environ Res Public Health. 2022 May 13;19(10):5966. doi: 10.3390/ijerph19105966 (PMC9141416; doi:10.3390/ijerph19105966)
Supplement: Supplementary file 1 [file ijerph-19-05966-s001.zip › ijerph-1722966-supplementary.pdf]

Restraint Use for Child Occupants in Dubai, United Arab Emirates

Codebook
SPSS variables

SPSS Variable name	Full variable name	Coding instructions	Measurement level
id	Identification number		1=<br>2=<br>3=<br>4=<br>5=
Age	age group	1=18-25 years<br>2=26-35 years<br>3=36-49 years<br>4=50-74 years<br>5=75 years and older	
Gender	gender	1=Male<br>2=Female	
Education	Highest level of education completed	1=None<br>2=Middle School<br>3=High School<br>4=University Graduate degree<br>5=University Post Graduate degree (e.g., Masters)	
Nationality	Nationality		
Residency	UAE resident	1=Yes<br>2=No	
Emirate	If UAE resident, which emirate		
Seats	Number of seats in vehicle		
Seatbelt	adult seatbelt use	1=Always<br>2=Almost always<br>3=Sometimes<br>4=Almost never<br>5=Never	

B. Use of restraints for child vehicle occupants

12	Number of children 0-10	
13	What is the age and gender of each of the children that you currently transport in your passenger vehicle?	Child 1: Age ___ years ___ months<br>Gender: □ Male □ Female<br>Child 2: Age ___ years ___ months<br>Gender: □ Male □ Female<br>Child 3: Age ___ years ___ months<br>Gender: □ Male □ Female<br>Child 4: Age ___ years ___ months<br>Gender: □ Male □ Female<br>Child 5: Age ___ years ___ months<br>Gender: □ Male □ Female<br>Child 6: Age ___ years ___ months<br>Gender: □ Male □ Female

Interviewers will Show the participant 'Restraint Type'

14	What kind of restraint does each of the children that you currently transport in your passenger vehicle use (choose from the restraint type pictures), and how often do they use this restraint?	Child 1: Restraint type: ___<br>□ Always □ Almost always □ Sometimes<br>□ Almost never □ Never<br><br>Child 2: Restraint type: ___<br>□ Always □ Almost always □ Sometimes<br>□ Almost never □ Never<br><br>Child 3: Restraint type: ___<br>□ Always □ Almost always □ Sometimes<br>□ Almost never □ Never<br><br>Child 4: Restraint type: ___<br>□ Always □ Almost always □ Sometimes<br>□ Almost never □ Never<br><br>Child 5: Restraint type: ___<br>□ Always □ Almost always □ Sometimes<br>□ Almost never □ Never<br><br>Child 6: Restraint type: ___<br>□ Always □ Almost always □ Sometimes<br>□ Almost never □ Never
15	What is the most important reason that your child is not 'always' restrained during passenger vehicle travel?	□ I don't have enough space in the vehicle<br>□ Child restraint systems or child seats are too expensive<br>□ My children feel uncomfortable or cry when restrained<br>□ Destinations are all close, only inside the city

		□ No legal penalty of not restraining child vehicle occupants it in Dubai<br>□ I don't believe it is important<br>□ Other reason(s): ________________________________<br>□ Not applicable, my child is 'always' restrained during passenger vehicle travel
16	Are there any laws in Dubai about restraining your children while travelling in a passenger vehicle?	□ No □ Yes

C. Choosing the car seat

17	Where did you get your car seat from?	□ Shop (in-store)<br>□ Shop (online)<br>□ Friend or relative<br>□ Other: ____________________<br>□ None, I do not use car seat
18	What information sources did you use to choose the car seat?	□ Word of mouth (e.g., friend or relative)<br>□ Car seat ranking websites<br>□ Official guidelines (e.g., RTA, Dubai Health Authority)<br>□ Sales people<br>□ Healthcare professionals (e.g., doctors, nurses)<br>□ Other: ____<br>□ Not applicable, I do not use a car seat
19	Did the information source include advice or instructions regarding the correct installation and use of the car seat?	□ No □ Yes<br>□ Not applicable, I do not use a car seat
20	How useful was the information source for the installation and use of the car seat?	□ Very useful □ Somewhat useful<br>□ Not at all useful<br>□ Not applicable, I do not use a car seat
21	When choosing your car seat, what was the most important reason for your selection?	□ Price<br>□ Brand name and popularity<br>□ Safety features<br>□ Recommendations from friends or relatives<br>□ Recommendations from sales people<br>□ Recommendations from healthcare professionals<br>□ Child's comfort<br>□ Other: ____<br>□ Not applicable, I do not use a car seat

D. Using restraints for child vehicle occupants

22	In your opinion, when should child vehicle occupants start using each of these restraint types?	1. Capsule/Infant seat: Start at: ______ years old<br>2. Child seat: Start at: ______ years old<br>3. Booster seat: Start at: ______ years old<br>4. Adult seatbelt: Start at: ______ years old	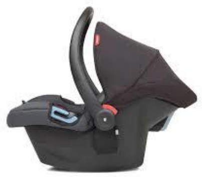<br>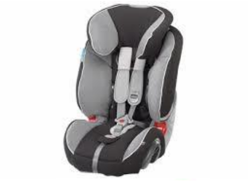<br>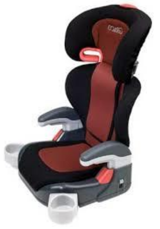<br>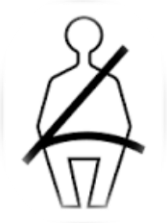
23	In your opinion, when should child vehicle occupants be allowed to sit in the front seat of the passenger vehicle?	Start at: ______ years old	

Thank you very much for completing this interview.

Your answers will be very helpful in improving our understanding about child vehicle occupant restraint use in Dubai.
